# Causes and Consequences of Flavivirus RNA Methylation

**DOI:** 10.3389/fmicb.2017.02374

**Published:** 2017-12-05

**Authors:** Shelton S. Bradrick

**Affiliations:** Department of Biochemistry and Molecular Biology, University of Texas Medical Branch, Galveston, TX, United States

**Keywords:** methylation, flavivirus, Zika virus, dengue virus, hepatitis C virus

## Abstract

Mosquito-borne flaviviruses are important human pathogens that represent global threats to human health. The genomes of these positive-strand RNA viruses have been shown to be substrates of both viral and cellular methyltransferases. N^7^-methylation of the 5′ cap structure is essential for infection whereas 2′-*O*-methylation of the penultimate nucleotide is required for evasion of host innate immunity. N^6^-methylation of internal adenosine nucleotides has also been shown to impact flavivirus infection. Here, I summarize recent progress made in understanding roles for methylation in the flavivirus life-cycle and discuss relevant emerging hypotheses.

## Introduction

The genomes of mosquito-borne flaviruses are complex, multi-functional RNA molecules that must be translated, replicated and packaged in the face of innate host defenses to accomplish the ultimate viral goal: production of infectious particles to initiate new rounds of infection. Viral genomes must interface with viral proteins and host machinery to accomplish these critical tasks. Such interactions are specified by RNA features within viral genomes, including sequences and secondary/tertiary structures, and *trans*-acting factors that recognize these *cis*-acting features (Campos et al., 2017). In addition to RNA sequence and structure, covalent modifications of individual nucleotides represent another layer of *cis*-acting features that have been shown to impact RNA function (Saletore et al., 2012). An RNA modification fundamental to flavivirus infection is methylation, as evidenced by the existence of virus-encoded RNA methyltransferases (MTase) (Dong et al., 2014). Moreover, a few recent studies implicate flavivirus genomes to be functionally methylated by host enzymes. In this review I summarize the current state of knowledge of flavivirus RNA methylation as well-effects of RNA methylation on flavivirus infection.

Multiple flaviviruses transmitted by arthropods represent serious human health concerns. These include yellow fever virus (YFV), West Nile virus (WNV), Zika virus (ZIKV), Japanese encephalitis virus (JEV) and the four serotypes of dengue viruses (DENV) which are the most prevalent, causing nearly 100 million symptomatic infections world-wide (Bhatt et al., 2013). These viruses, comprising part of the flavivirus genus, belong to the *Flaviviridae* which includes the significant blood-borne human pathogen within the hepacivirus genus, hepatitis C virus (HCV). The genomes of viruses within this family share a similar organization: each contains a single open reading frame flanked by untranslated regions (UTRs) of various sequence, length and structure. The viral UTRs contain functional RNA elements that control viral translation and RNA synthesis (Garcia-Blanco et al., 2016). Unique to members of the flavivirus genus is the presence of a so called “cap” structure at the 5′ end of the genome. As discussed in detail below, methylation of the cap structure and the adjacent penultimate nucleotide of the viral genome critically promotes virus infection by multiple mechanisms. In contrast, HCV, the most prominent member of the hepacivirus genus, is characterized by an uncapped genome that contains an internal ribosome entry site within the 5′ UTR (Tsukiyama-Kohara et al., 1992).

At the level of the individual cell all *Flaviviridae* use a fundamental infection strategy: (i) virus particles attach to various cellular receptors and are internalized via endocytosis, (ii) endosome acidification causes fusion between the viral envelope and endosomal membrane allowing for escape of the viral nucleocapsid into the cytoplasm, (iii) the viral RNA dissociates from capsid and engages the translational machinery to synthesize viral proteins at the cytosolic face of the endoplasmic reticulum (ER), (iv) viral proteins engage the positive-strand genome to synthesize a negative strand intermediate, (v) the negative-strand asymmetrically templates the synthesis of many genomes, (vi) some of which associate with viral structural proteins and bud into the ER to form immature viral particles that (vii) transit through the golgi apparatus where they are modified by host enzymes, and finally, (viii) mature virions are secreted into the extracellular space. Note that multiple phases of the life-cycle, including translation, RNA synthesis and virus assembly, occur concurrently on separate genomes once infection is established although completion of each process is a prerequisite for the following to occur.

## Roles for Methylation At the 5′ End of the Flavivirus Genome

**FIGURE 1:**
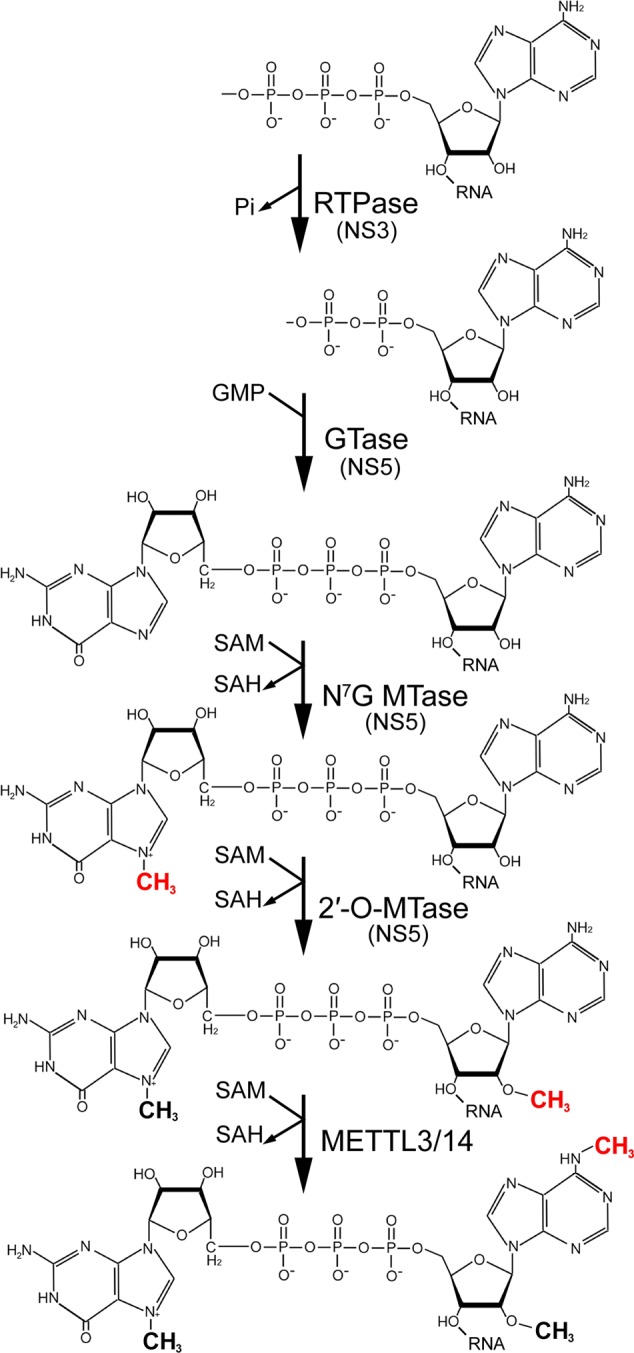
Depiction of the flavivirus RNA capping and methylation pathway. Nascent flavivirus genomes initiate with a 5′ triphosphorylated adenosine that is dephosphorylated by the RNA triphosphatase (RTPase) activity of NS3. Next, the putative NS5 guanylyltransferase (GTase) attaches guanosine monophosphate (GMP) via a 5′-5′ linkage. NS5 then methylates the guanine N^7^ position to form the type 0 cap using *S*-adenosyl methionine (SAM) as a cofactor. Methyl group donation by SAM converts it to *S*-adenosyl homocysteine (SAH). NS5-mediated 2′-*O*-methylation of the adenosine nucleotide generates the type I cap structure. Finally, hypothetical m^6^A methylation of flaviviral RNA at the penultimate adenosine by the METTL3/14 protein complex would result in the formation m^6^Am.

Cellular mRNAs are modified in the nucleus with a 7-methylguanosine (m^7^GpppN) cap structure attached to the first base of the transcript via a 5′-5′ triphosphate linker (**Figure 1**; Shatkin, 1976). This occurs early after the initiation of transcription via recruitment and sequential action of capping enzymes, including an RNA triphosphatase, guanylyltransferase and N^7^-guanine MTase, to the C-terminal domain of elongating RNA pol II (Phatnani and Greenleaf, 2006). The RNA triphosphatase acts to remove the γ-phosphate from the 5′ nucleotide of the nascent RNA making it available for cap addition by guanylyltransferase. Methylation of the guanosine cap at N^7^ completes the reaction to generate a so called “type 0” cap structure (Wei C. M. et al., 1975). Importantly, in higher eukaryotic organisms the mRNA is further modified by a separate ribose MTase at the penultimate nucleotide with a 2′-*O*-methyl group (**Figure 1**; type 1 cap) and to a lesser extent at the following nucleotide (type 2 cap) (Wei and Moss, 1975). The 5′ cap structure impacts every aspect of mRNA metabolism, including splicing, nuclear export, translation, and decay (Cowling, 2010). In contrast, 2′-*O*-methylation is a mark that signifies an mRNA as a “self” versus foreign molecule.

Flaviviruses do not have access to the nuclear m^7^G-capping machinery and instead have evolved enzymatic activities to carry out all the necessary steps to generate capped genomes. The NS5 protein, in addition to its essential RNA-dependent RNA-polymerase function, harbors guanylyltransferase and MTase enzymes (Egloff et al., 2002; Ray et al., 2006; Issur et al., 2009). NS3 is similarly multifunctional, capable of protease, helicase and RNA triphosphatase activities (Wengler and Wengler, 1993; Bartelma and Padmanabhan, 2002). The latter of these catalyze the first step in capping: removal of the γ-phosphate from the 5′ adenosine of nascent viral RNAs. NS5 is then believed to cap the RNA and performs sequential methylation reactions to generate (i) m^7^GpppA (cap-0) and then (ii) m^7^GpppAm (cap-1) (Ray et al., 2006). The *cis*- and *trans*-determinants of putative guanylyltransferase activity have not been well-characterized but the MTase reactions are relatively well-understood. Unlike cellular MTase which is not believed to discriminate among different RNA substrates, the flavivirus MTase exhibits substrate specificity and will not efficiently methylate the cap of non-viral RNAs (Dong et al., 2007). Cap methylation by NS5 requires the second and third genome nucleotides to be GU and also the presence of a 5′ stem loop which is structurally conserved across all flaviviruses (Brinton and Dispoto, 1988). 2′-*O*-methylation requires the first two nucleotides (AG) and is enhanced by sequence within the first 20 residues of the genome. Notably, m^7^GpppA-RNA is strongly preferred over GpppA-RNA as a substrate for 2′-*O*-methylation, explaining the sequential order of 5′ end methylation reactions (Dong et al., 2008).

What are the functional consequences of cap and 2′-*O*-methylation? Mutational analyses of the NS5 MTase have identified residues that specifically ablate 2′-*O*-methylation, cap-methylation, or both. Interestingly, loss of 2′-*O*-methylation can be tolerated whereas cap methylation is essential for infection (Zhou et al., 2007; Dong et al., 2010). In considering why cap methylation is critical, it is worthwhile to consider data from Ray et al. (2006) who measured the effects of GpppA, m^7^GpppA and m^7^GpppAm caps on translation and RNA synthesis using the WNV replicon system which encodes the *Renilla* luciferase (RLuc) reporter. Compared to uncapped (pppA) WNV replicon RNA, the addition of GpppA strongly (∼25-fold) enhanced the accumulation of (RLuc) at 2 h post-transfection. Surprisingly, m^7^GpppA enhanced replicon RNA translation by only twofold compared to unmethylated GpppA cap, and m^7^GpppAm did not further increase RLuc expression. Unexpectedly, no differences in replicon RNA levels were detected for the differently capped replicons. By 72 h post-transfection, each of the capped replicon RNAs produced similar RLuc, indicating that methylation is not required for initial negative-strand synthesis as this is a prerequisite for synthesis of downstream positive-strand synthesis and consequent production of RLuc.

There are at least three non-mutually exclusive explanations for a cap methylation requirement by flaviviruses. First, the cap structure itself is known to protect RNA from 5′ to 3′ exonucleases such as Xrn1 (Hsu and Stevens, 1993) and likely plays a significant role in preventing viral RNA decay. However, it is not clear whether cap methylation plays a significant role in stabilizing RNA. Indeed, the human decapping protein Dcp2 cannot act on an capped RNA substrate lacking N^7^-methylation (Wang et al., 2002), suggesting that methylation actually enables decapping which is a prerequisite for 5′-3′ decay (Wilusz et al., 2001). Nevertheless, it is possible that cap methylation may render flaviviral genomes resistant to cellular RNases by an unknown mechanism.

A role in stimulating viral translation initiation is a plausible explanation for a cap methylation requirement by flaviviruses. The canonical mRNA cap-binding protein, eIF4E, is believed to be essential for translation of most cellular mRNAs through indirect recruitment of the 40S ribosomal subunit and associated initiation factors (Hershey et al., 2012). EIF4E strongly discriminates between m^7^Gppp and Gppp, and early studies by Shatkin and colleagues demonstrated that methylation enhanced cap-dependent translation (Both et al., 1975; Muthukrishnan et al., 1975). There are, however, a few clues that flavivirus translation initiation may occur via a non-canonical mechanism. First, as noted above cap methylation conferred a relatively small translational advantage to WNV replicon RNAs (Ray et al., 2006). Second, depletion of eIF4E by RNA interference was reported to not affect DENV replication or protein synthesis: whereas DENV translation was reduced by ∼10%, cellular translation was reduced by 60% due to eIF4E knockdown (Edgil et al., 2006). These observations suggest that flaviviruses may use a non-canonical pathway of translation initiation that depends minimally on the presence of a 5′- m^7^Gppp.

Finally, it is hypothetically possible that cap methylation protects viral genomes from recognition by factor(s) that sense unmethylated cap structures as invading nucleic acid. No such factor has yet been identified but there are many well-defined “pattern recognition receptors” whose tasks are to detect invading non-self nucleic acids and directly or indirectly, through innate immune pathways, antagonize infection (Wu and Chen, 2014). A pertinent example of these factors is the IFIT family of proteins discussed below.

What are the functional consequences of 2′-*O*-methylation at the penultimate nucleotide? As noted above, loss of this methylation event does not cause virus lethality in contrast to cap methylation. Key insights into this question were made by Diamond and colleagues who observed that a 2′-*O*-methylation-deficient NS5 mutant (E218A) WNV lacking m^7^GpppAm was attenuated in immunocompetent mice and primary cells, whereas animals and cells lacking the type I interferon (IFN) receptor (IFNAR-/-) were fully susceptible to infection (Daffis et al., 2010). These authors went on to show that IFN-inducible proteins of the IFIT family (murine IFIT1 and IFIT2) disproportionately restricted WNV lacking 2′-*O*-methylation compared to WT virus. Some IFIT proteins have been described to inhibit translation via binding and interfering with the function of eIF3 (Hui et al., 2003), a complex of initiation factors that recruit the 40S ribosomal subunit to mRNA via interaction with eIF4G during initiation of translation (Hershey et al., 2012). More recently, several groups have identified human IFIT1 as a protein that binds directly to cap-0 and blocks translation (Kumar et al., 2014; Abbas et al., 2017), presumably by hindering access of eIF4E to the cap structure. This translational suppression coincides with an accelerated innate immune response that compromises infection (Schmid et al., 2015; Chang et al., 2016). Taken together, these reports strongly suggest that 2′-*O*-methylation of the cap is an epigenetic RNA modification that allows cells to differentiate self versus non-self RNAs via IFIT proteins. Clearly flaviviruses, and indeed many other types of viruses, have evolved mechanisms to evade IFIT-mediated restriction by encoding their own 2′-*O*-MTases.

## Roles for Internal Adenosine Methylation In Infection By Flaviviruses and HCV

It has been recognized for several decades that a prominent modification to cellular mRNA across many diverse organisms is the methylation of adenosine at the N^6^ position (Desrosiers et al., 1974; Perry and Kelley, 1974; Zhao et al., 2016). This occurs at internal mRNA positions (m^6^A) and also at the penultimate nucleotide of transcripts that initiate with A (**Figure 1**). The latter is referred to as m^6^Am as it is also methylated at the 2′-hydroxyl (Wei C. et al., 1975). In the past few years research on m^6^A has greatly expanded and multiple studies have addressed roles of m^6^A in virus infection. Several methyltransferase and demethylase enzymes have been identified as well as proteins that can recognize methyl groups in RNA (Zhao et al., 2016; Meyer and Jaffrey, 2017). These factors are referred to as “writers,” “erasers,” and “readers” of m^6^A. A key recent innovation is the use of m^6^A-specific antibodies in RNA-immunoprecipitation to allow transcriptome-wide mapping of m^6^A locations in RNA molecules (Dominissini et al., 2012; Meyer et al., 2012; Linder et al., 2015). This has enabled identification and functional analysis of m^6^A sites by mutation of the low complexity consensus motif DRACH (D = G/A/U; R = G/A; H = C/A/U).

To date m^6^A mapping and some functional analyses have been performed on multiple viruses including influenza A virus (Courtney et al., 2017), human immunodeficiency virus (Kennedy et al., 2016; Lichinchi et al., 2016a; Tirumuru et al., 2016), HCV (Gokhale et al., 2016), YFV (Gokhale et al., 2016), DENV (Gokhale et al., 2016), WNV (Gokhale et al., 2016), and ZIKV (Gokhale et al., 2016; Lichinchi et al., 2016b). Relevant to this discussion are the studies conducted on *Flaviviridae* by Gokhale et al. (2016) and Lichinchi et al. (2016b) who characterized functional roles of m^6^A in HCV and ZIKV infection, respectively. To determine the effects of m^6^A on infection, Gokhale et al. (2016) depleted the key methylase (METTL3 plus its co-factor METTL14) and demethylases (FTO and ALKBH5) by RNA interference and assayed effects on HCV infection. Intriguingly, knockdown of METTL3/14 enhanced infection while FTO depletion correspondingly reduced infection. These results are consistent with an antiviral role for m^6^A in the HCV life-cycle. Notably, depletion of these enzymes had no effect on HCV translation or RNA synthesis, suggesting a role for m^6^A in opposing a late stage of infection such as assembly or egress of infectious virus. Consistent with this idea, several known cytosolic reader proteins (YTHDF1-3) suppressed viral titers, co-immunoprecipitated HCV RNA and localized to lipid droplets which are known sites of HCV assembly (Miyanari et al., 2007). Silent mutation of four m^6^A sites within the envelope coding region enhanced infection, providing further evidence for a restrictive role of m^6^A in HCV infection. Gokhale et al. (2016) went on to map m^6^A in the genomes of multiple mosquito-transmitted flaviviruses, including DENV, YFV, WNV, and two divergent strains of ZIKV. Of note, this analysis revealed abundant m^6^A within the NS5 coding regions of these viruses.

In their companion article to the Gokhale et al. (2016) study, Lichinchi et al. (2016b) and colleagues mapped locations m^6^A on ZIKV RNA and investigated the roles of readers, writers and erasers in infection. Depletion of METTL3 or METTL14 enhanced ZIKV infection in 293T cells whereas ALKBH5 and, to a lesser extent, FTO knockdown reduced infection. Moreover, YTDHF1/2 expression negatively correlated with ZIKV RNA levels released from infected cells, suggesting antagonism of ZIKV infection by these reader proteins and YTHDF2 in particular. The authors speculated that YTHDF2 may bind to and destabilize ZIKV RNA. Finally, Lichinchi et al. (2016b) reported that ZIKV infection alters the host m^6^A methylome, implying that gene expression changes caused by infection may be partly due to altered m^6^A patterns on cellular mRNA.

Many open questions remain in the nascent field addressing roles for m^6^A in flavivirus infection. What is the mechanism by which m^6^A inhibits ZIKV infection? Are similar effects and modes of action operating during infection with other flaviviruses? Does m^6^A impact cellular innate immune responses? How does infection impact functionality of the machinery that regulates the m^6^A methylome? Does m^6^A control flavivirus infection *in vivo*? In the context of HCV, how do YTHDF proteins suppress late stages of the virus life-cycle?

Lichinchi et al. (2016b) observed elevated titer and viral RNA in supernatants of cells with reduced levels of METTL3/METTL14 which implies that m^6^A modification opposes virus infection. It should be noted that the overall effects on ZIKV production may be considered mild (< ∼2-fold), indicating that m^6^A acts as a moderate restriction factor for infection *in vitro*. Nevertheless, the relatively small effects could reflect a viral strategy that is able to counter, in part, otherwise potent restriction by m^6^A and *trans*-acting reader proteins. One hypothesis is that the subgenomic flaviviral RNA (sfRNA), a highly stable RNA fragment produced from decay of virus genomes (Pijlman et al., 2008), could act to buffer the negative impact of m^6^A by sequestering YTHDF reader proteins. It would be of interest, for example, to test whether strains of DENV that produce different amounts of sfRNA would be differentially susceptible to inhibition by m^6^A (Manokaran et al., 2015). Of course, this is only one hypothesis and there remains much work to be done to gain a thorough understanding of how flavivirus infections are affected by m^6^A.

## Conclusion

Flavivirus RNA methylation critically impacts infection. Cap methylation (m^7^GpppA) is essential for infection, at least in part due to its role in stimulating virus translation. Methylation at the 2′-hydroxyl of the penultimate adenosine (m^7^GpppAm) is inessential for viability but allows the virus to escape the inhibitory actions of IFIT proteins and likely other factor(s) (Szretter et al., 2012). In contrast, internal m^6^A modifications are somewhat deleterious to ZIKV and HCV infections *in vitro* although there is much to be learned regarding roles for m^6^A in infection. Studies addressing viral RNA methylation are informative with respect to basic virus biology but may also allow development of approaches to control infections by pathogenic flaviruses for which there are no currently available therapeutics. Drugs specifically targeting flavivirus MTase enzymes could be potent antivirals for the treatment of patients with acute infections. Moreover, mutant flaviviruses lacking 2′-*O*-MTase activity have shown promise as candidate vaccine strains because they are attenuated yet induce robust immunity to heterologous infection with virulent viruses (Li et al., 2013; Züst et al., 2013). Thus, understanding the basic molecular mechanisms of flavivirus biology will hopefully lead to measures that reduce the burden caused by these viruses.

## Author Contributions

The author confirms being the sole contributor of this work and approved it for publication.

## Conflict of Interest Statement

The author declares that the research was conducted in the absence of any commercial or financial relationships that could be construed as a potential conflict of interest.
